# Vermiculite, Respiratory Disease, and Asbestos Exposure in Libby, Montana: Update of a Cohort Mortality Study

**DOI:** 10.1289/ehp.9481

**Published:** 2007-01-03

**Authors:** Patricia A. Sullivan

**Affiliations:** Field Studies Branch, Division of Respiratory Disease Studies, National Institute for Occupational Safety and Health, Morgantown, West Virginia, USA

**Keywords:** amphibole fibers, asbestos, asbestosis, asbestos-related disease, insulation, lung cancer, mesothelioma, richterite, tremolite, winchite

## Abstract

**Background:**

Vermiculite from the mine near Libby, Montana, is contaminated with tremolite asbestos and other amphibole fibers (winchite and richterite). Asbestos-contaminated Libby vermiculite was used in loose-fill attic insulation that remains in millions of homes in the United States, Canada, and other countries.

**Objective:**

This report describes asbestos-related occupational respiratory disease mortality among workers who mined, milled, and processed the Libby vermiculite.

**Methods:**

This historical cohort mortality study uses life table analysis methods to compare the age-adjusted mortality experience through 2001 of 1,672 Libby workers to that of white men in the U.S. population.

**Results:**

Libby workers were significantly more likely to die from asbestosis [standardized mortality ratio (SMR) = 165.8; 95% confidence interval (CI), 103.9–251.1], lung cancer (SMR = 1.7; 95% CI, 1.4–2.1), cancer of the pleura (SMR = 23.3; 95% CI, 6.3–59.5), and mesothelioma. Mortality from asbestosis and lung cancer increased with increasing duration and cumulative exposure to airborne tremolite asbestos and other amphibole fibers.

**Conclusions:**

The observed dose-related increases in asbestosis and lung cancer mortality highlight the need for better understanding and control of exposures that may occur when homeowners or construction workers (including plumbers, cable installers, electricians, telephone repair personnel, and insulators) disturb loose-fill attic insulation made with asbestos-contaminated vermiculite from Libby, Montana.

Vermiculite is a naturally occurring mineral mined in the United States, Brazil, Argentina, Mexico, South Africa, Zimbabwe, Kenya, Uganda, Egypt, India, Russia, China, Japan, and Australia. Mined vermiculite ore is milled to produce vermiculite concentrate of various sizes and grades. When rapidly heated, vermiculite concentrate expands to form small, light-weight, accordion-shaped granules. Vermiculite is used in construction products (loose-fill attic insulation, acoustic finishes, spray-on fireproofing, gypsum plaster, concrete mixes for swimming pools), consumer products (packing materials, adsorbent in laboratories), agricultural and horticultural products (animal feed, bulking agent, fertilizers, pesticides, seed encapsulant, hydroponics, potting mixes, soil conditioners), and in industrial products (brake shoes and pads, drilling muds, furnaces, filters, insulator blocks, paints, and sealants) [U.S. Environmental Protection Agency (EPA) 2006].

Vermiculite from the mine that operated near Libby, Montana, from the early 1920s until 1990 was contaminated with asbestos and other fibrous amphibole minerals, crystalline silica, and talc. The U.S. Geological Survey has characterized the respirable fraction of asbestiform amphiboles contaminating the Libby vermiculite as approximately 84% winchite, 11% richterite, and 6% tremolite (Meeker et al. 2003). The raw Libby ore was estimated to be 21–26% asbestos by weight; the mill feed was 3.5–6.4% asbestos; airborne dust in the dry mill was 40% asbestos (Wake 1962); and the vermiculite concentrate shipped to numerous processing plants in the United States and other countries was 0.3–7.0% asbestos before expansion (Amandus et al. 1987; Atkinson et al. 1981).

Previous studies of Libby workers documented increased risk of lung cancer and nonmalignant respiratory disease among highly exposed workers with at least 1 year tenure (Amandus and Wheeler 1987; McDonald et al. 1986, 2004). Reports of respiratory disease mortality among community residents and household contacts of Libby vermiculite workers suggested increased risk from transient exposure or ambient community exposure [Agency for Toxic Substances and Disease Registry (ATSDR) 2002; Schneider 1999]. Cross-sectional radiographic screening conducted in Libby for the ATSDR revealed that 6.7% of community residents with no occupational or familial exposure have radiographic evidence of asbestos-related disease (Peipins et al. 2003). These findings suggest that risk from asbestos-contaminated vermiculite may not be limited to those with high-intensity occupational exposure.

This report expands the previously studied occupational cohort to include all white men hired at Libby from September 1935 through December 1981. The intent here is to describe the mortality experience of workers exposed to Libby amphibole fibers (tremolite asbestos, winchite, richterite) over the full range of exposure and employment duration. Occupational respiratory disease mortality among Libby workers is compared to that expected based on the mortality experience of the U.S. population, and standardized mortality ratios (SMRs) and standardized rate ratios (SRRs) for asbestosis, lung cancer, and all nonmalignant respiratory diseases are presented.

## Materials and Methods

### Study subjects

Study subjects were vermiculite miners, millers, and processors. Workers also may have been assigned jobs in the screening plant, railroad loading dock, expansion plants, or an office located in the town of Libby (several miles from the mine). The cohort was enumerated in May 1982, and study subjects were followed through December 2001. The design allowed a minimum 20 years of follow-up since first exposure, with > 65 years of follow-up for the earliest hired workers. Demographic and work history data were abstracted from company personnel and pay records. A database created by the National Institute for Occupational Safety and Health (NIOSH) in the 1980s contained demographic data, work history, and vital status at the end of 1981 for 1,881 workers. The data were validated against company records on microfilm at NIOSH, and work history data were reabstracted. One person was removed from the cohort because company records stated that he was hired but never worked. Nine workers with Social Security numbers listed in company records were excluded because demographic and work history data were not available, leaving 1,871 potential study subjects.

### Vital status follow-up

The NIOSH Institutional Review Board approved the research protocol; the study complied with all applicable U.S. requirements and regulations for studies involving human subjects. Vital status follow-up through 2001 used the National Death Index (NDI-Plus), the Social Security Administration, the Internet [Ancestry.com (2006), RootsWeb.com (2006), and electronic links to state death records], and a tracing service. Workers known to be alive on or after 1 January 1979 (the date NDI began tracking deaths nationwide), but not found in the NDI, were assumed to be alive at the study end date of 31 December 2001. Vital status follow-up was completed for 97.8% of the cohort (*n* = 1,830). Nearly 47% of these workers (*n* = 877) had died by 31 December 2001.

For 97% of those known to be deceased, cause of death was determined from death certificates and coded to the *International Classification of Diseases* (ICD) using the rubrics of the ICD revision in effect at the time of death {ICD-8 (National Center for Health Statistics 1967); ICD-9 [World Health Organization (WHO) 1977]; or ICD-10 (WHO 1992)}. Deaths before 1979 were coded by a single National Center for Health Statistics-trained nosologist; for 1979–2001, ICD codes were obtained from the NDI.

### Exposure assessment

The mining, milling, and processing operations at Libby; conditions of exposure; and job-specific estimates of exposure intensity have been thoroughly described previously (Amandus and Wheeler 1987; Amandus et al. 1987, 1988; McDonald et al. 1986). Briefly, miners extracted vermiculite ore from an open-pit mine. The ore was processed in a dry mill (1935–1976) and/or two wet mills (1955–1990) that operated on Vermiculite Mountain. The resulting concentrate was shipped by railroad to processing plants where the vermiculite was expanded for use in loose-fill attic insulation. Before 1975, exposures in the mine ranged from 9–23 fibers per cubic centimeter (fibers/cc) of air for drillers; exposures in other mining jobs were estimated to be < 2 fibers/cc (Amandus et al. 1987). Early fiber exposures in the dry mill were as high as 182 fibers/cc during sweeping operations; by 1964, exposures in the mill had been reduced by 80%. Amandus et al. (1987) estimated that by 1972 exposures in all work areas were < 1 fiber/cc as an 8-hr time-weighted average, compared with today’s Occupational Safety and Health Administration (OSHA) asbestos standard of 0.1 fiber/cc (U.S. Department of Labor 2006).

The job-exposure matrix developed for this study was based on that used in the earlier NIOSH study (Amandus and Wheeler 1987), with some important exceptions. In the earlier study, workers with “common laborer” job assignments and some workers with unknown job assignments were assigned the relatively low exposure estimated for the mill yard. In the current analysis, these workers were assigned the average estimated exposure intensity for all unskilled jobs during the relevant calendar time period. This resulted in higher estimates of cumulative exposure, with likely attenuation of the effect estimators. In addition, reabstracting work histories for the current study identified several job assignments not mentioned in the earlier publications. Exposure estimates for the additional job/calendar time period combinations were extrapolated based on professional experience and review of exposure records from earlier studies of Libby workers (Amandus and Wheeler 1987; Amandus et al. 1987, 1988; McDonald et al. 1986).

The exposure index available for this and previous studies of Libby workers is based on fiber count data obtained using optical phase contrast microscopy (PCM). PCM measures fibers longer than 5 μm and wider than 0.25 μm [i.e., the fiber size regulated under the OSHA standard (U.S. Department of Labor 2006)]. Fiber count data obtained using PCM do not distinguish between the various amphiboles in the Libby vermiculite. Thus, fiber count estimates used in the exposure assessment include not only the regulated tremolite asbestos fibers but also the asbestiform amphibole fibers not mentioned in the regulations (winchite and richterite). Recall that Meeker et al. (2003) characterized the Libby amphibole as 6% tremolite. If the observed health effects are explained by tremolite asbestos alone, then exposure has been considerably overestimated, and the effect of each fiber per cubic centimeter-year increment in exposure has been substantially underestimated (McDonald et al. 2004).

### Treatment of missing values

Dates of termination were unknown for 58 of 640 workers (9%) who left employment before September 1953. These workers were assumed to have worked for 384 days, based on the mean duration of employment among all workers with known termination date before September 1953. U.S. Census Bureau data (2004) indicate that 95% of the local population identify themselves as white. Because workers at this facility were known to be primarily Caucasian, 935 workers with race unknown were assumed to be white (NIOSH 2001). Similarly, because 96% of the workforce was male, seven workers with sex unknown were assumed to be male after review of names.

### Statistical analysis

Data were managed using ACCESS 2000 (Microsoft Corporation, Redmond, WA) and SAS, Version 8.0 (SAS Institute, Cary, NC). Descriptive analyses were performed using SAS, Version 9.1 (SAS Institute).

Because 95% of study subjects were white men, the analytic cohort was limited to 1,672 white males [excluding 95 men who died or were lost to follow-up before 1960, the first-year for which comparison rates for asbestosis are available in the NIOSH Life Table Analysis System (LTAS) (NIOSH 2001)]. Using LTAS software (NIOSH 2001; Steenland et al. 1990, 1998), SMRs were calculated to determine if study subjects experienced greater mortality from specific causes than was expected based on the U.S. population experience. SMRs were adjusted for age at risk and calendar year of follow-up (categorized into 5-year age and calendar time groups). SRRs evaluated exposure response across increasing categories of cumulative exposure and duration of employment, with workers in the lowest exposure group serving as the baseline for comparison. A formal test for a linear trend in the slope of the SRRs evaluated the hypothesis that β_1_ = 0 against the alternative hypothesis that duration or cumulative exposure predicts mortality. The 95% confidence limits were calculated. If the 95% confidence interval (CI) for the slope did not include zero, the hypothesis was rejected in favor of the alternative at the *p* < 0.05 level.

Cumulative exposure data were categorized to achieve an approximately equal number of cases in each exposure category, a method previously found to be most efficient (Richardson and Loomis 2004; Sullivan et al. 1996), while maintaining sufficient person-years in each exposure category to obtain valid estimators. In some analyses, the lowest categorical cut point was set at 4.5 fibers/cc-years [i.e., a worker’s cumulative lifetime exposure if exposed to asbestos fibers at the current OSHA standard of 0.1 fibers/cc over a 45-year working life (U.S. Department of Labor 2006)]. Exposure duration was categorized to facilitate meaningful interpretation (i.e., < 1 year, 1–9.9 years, ≥ 10 years). The lag period was chosen to avoid excluding cases with disease assumed to be work-related. For the outcomes of interest, I found an exposure lag of 15 years to present a clear picture of exposure response.

Person-years at risk and observed deaths were accrued from the date comparison rates were available (1 January 1960), or from the date of first exposure (if later) until the time that each worker died or was lost to follow-up, or until the end of the study (31 December 2001). Ten workers lost to follow-up were considered to be alive, but person-years of observation were truncated on the date each of these workers was last observed alive (date of termination, or last date known to be alive in earlier vital status follow-up). One worker was excluded from the SMR analysis because he died in Canada; another was excluded because his date of death was unknown. Twenty-four workers known to have died, but with cause of death unknown, were added to the residual cause code (NIOSH 2001).

The analysis presented here focuses on occupational respiratory conditions potentially related to asbestos exposure: lung cancer, asbestosis, and other nonmalignant respiratory disease. Mesothelioma was not coded as a distinct cause of death under ICD coding rubrics until 1999, so the SMR for mesothelioma is based on only 3 years of data (1999–2001).

## Results

Demographic and exposure characteristics of study subjects by selected causes of death are presented in Table 1. Among the 752 white men with known cause of death, 13.2% died from lung cancer, 2.0% from mesothelioma, and 5.3% with asbestosis. The average age at hire among study subjects was 29.7 years (range, 15.4–69.8 years). The mean duration of employment for all 1,672 study subjects was 4.0 years and ranged from 1 day to 43.1 years. In contrast, the mean duration of employment was 7.1 years among workers who died with cancer of the lung or bronchus, 10.8 years among those with mesothelioma, and 14.6 years among those with a diagnosis of asbestosis listed on their death certificates. Similarly, median cumulative exposure was estimated at 8.7 fibers/cc-years among all workers and 21.0 fibers/cc-years among those dying through 2001, but 28.2, 145.1, and 228.4 fibers/cc-years among those dying with lung cancer, mesothelioma, or asbestosis, respectively. On average, 34.8 years passed between hire and the study end date (or death); the maximum time since hire was 66.8 years.

Previous studies of this cohort included only workers employed for ≥ 1 year. Comparison of demographic characteristics between those who worked < 1 year and those who worked longer (not shown in Table 1) suggests that, initially, there was little difference between these groups, except with respect to age at hire. Perhaps because a number of students worked summers at Libby, those who left employment after < 1 year were younger at hire than those who worked longer (28.9 vs. 30.4 years; 2-sided *p* = 0.0016). There were, however, substantial differences between short-term and long-term workers with respect to occupational exposure. Short-term workers were employed for an average of 3 months, compared with 7.7 years among those who worked ≥ 1 year, and experienced lower cumulative exposure (median, 2.6 vs. 43.4 fibers/cc-years).

Allowing for a 15-year exposure lag, asbestos-exposed Libby vermiculite workers were 24% more likely to have died by the end of 2001 compared with white men of the same 5-year age group in the U.S. population (SMR = 1.2; 95% CI, 1.1–1.3), and were 37% more likely to have died from cancer (SMR = 1.4; 95% CI, 1.2–1.6). Libby workers also experienced significant excess mortality from cancer of the trachea, bronchus, and lung (SMR = 1.7; 95% CI, 1.4–2.1) and nonmalignant respiratory disease (SMR = 2.4; 95% CI, 2.0–2.9) after allowing for a 15-year exposure lag (Table 2).

Of the 15 mesothelioma deaths (1979–2001) identified by reviewing death certificates (Table 1), 1 worker died from peritoneal mesothelioma, and 14 died from pleural (or unspecified) mesothelioma. The SMR for mesothelioma (Table 2), based on the 2 deaths that occurred in 1999–2001, was 15.1 (95% CI, 1.8–54.4). Excess mortality was also observed for several conditions to which ICD coding rubrics assigned mesothelioma deaths before 1999. For example, there was a significant excess in mortality from cancer of the pleura (SMR = 23.3; 95% CI, 6.3–59.5) and in the LTAS minor category described as “malignancy of other and unspecified sites” (SMR = 2.4; 95% CI, 1.6–3.6). Similarly, there were 4 deaths from connective tissue cancer between 1940 and 2001, resulting in a statistically significant SMR of 4.7 (95% CI, 1.3–12.0; no lag). Lagged estimators for connective tissue cancer are not presented because small numbers likely result in unstable estimators.

Libby workers experienced significant excess mortality from asbestosis and other nonmalignant respiratory diseases (Table 2). After allowing for a 15-year exposure lag, the asbestosis SMR was 165.8 (95% CI, 103.9–251.1). Mortality ratios were elevated for chronic obstructive pulmonary disease (SMR = 2.2; 95% CI, 1.7–2.9) and the LTAS minor category described as “other respiratory diseases” (SMR = 2.7; 95% CI, 1.6–4.2).

Although this report is focused on occupational respiratory disease, an *a priori* goal of this study was to evaluate the cohort’s mortality from other potentially asbestos-related conditions, such as circulatory disease and digestive cancer. Libby workers experienced no overall excess in heart disease (SMR = 0.9; 95% CI, 0.8–1.1), but they did experience excess mortality from circulatory diseases involving the arteries, veins, and lymphatic vessels (SMR = 1.8; 95% CI, 1.2–2.6; 29 observed vs. 16 expected; ICD-9 codes 415–417, 440–459). Although not statistically significant, Libby workers employed for ≥ 1 year experienced excess mortality from cancer of the liver, gallbladder, or bile ducts (SMR = 1.6; 95% CI, 0.3–4.6; 3 observed vs. 1.89 expected; ICD-9 codes 155–156) and pancreatic cancer (SMR = 1.8; 95% CI, 0.7–3.8; 7 observed vs. 3.83 expected; ICD-9 code 157).

### Effect of cumulative exposure

Table 3 presents the evaluation of exposure–response relationships for asbestos-related occupational respiratory disease mortality. The SMR for lung cancer rose from 1.5 (95% CI, 0.9–2.3) among workers with < 4.5 fibers/cc-years cumulative exposure to 1.9 (95% CI, 1.2–2.9) among workers exposed to at least 100 fibers/cc-years (allowing for a 15-year exposure lag).

There was significant excess mortality from nonmalignant respiratory disease even among workers with < 4.5 fibers/cc-years cumulative exposure (SMR = 1.8, 95% CI, 1.1–2.8). The SMR for nonmalignant respiratory disease rose to 4.8 (95% CI, 3.1–7.3) among workers exposed to ≥ 300 fibers/cc-years (Table 3).

Cumulative exposure was a significant predictor of nonmalignant respiratory disease mortality even among those who worked < 1 year, with the SMR rising from 1.9 (95% CI, 1.1–3.2) among those with < 3.5 fibers/cc-years exposure to 2.6 (95% CI, 1.5–4.3) among short-term workers exposed to ≥ 15 fibers/cc-years (not shown). The test for a linear trend in the SRRs was statistically significant with *p* < 0.001.

Although 40 white male workers died with asbestosis listed on their death certificates (Table 1), SMR analysis is based on the 22 workers with asbestosis listed as the underlying cause of death (currently, the LTAS software does not include multiple cause comparison rates for asbestosis). SMRs for asbestosis increased with increasing cumulative exposure (Table 3). The SMR rose from 37.3 (approximate 95% CI, 7.5–122.3) among workers with < 50 fibers/cc-years exposure, to 749.1 (95% CI, 373.0–1367.8) among those with ≥ 250 fibers/cc-years cumulative exposure, after allowing for a 15-year exposure lag.

Table 3 also provides SRRs for lung cancer, nonmalignant respiratory disease, and asbestosis over increasing categories of cumulative exposure. For each outcome, linear trend tests were statistically significant at the *p* < 0.01 level.

### Effect of exposure duration

Those working < 1 year experienced a significant excess in lung cancer (SMR = 1.6; 95% CI, 1.1–2.1), with the SMR rising to 2.5 (95% CI, 1.4–4.3) among those working for ≥ 10 years (Table 4). The SMR for nonmalignant respiratory disease was 2.1 (95% CI, 1.6–2.8) among those who worked < 1 year, and rose to 3.6 (95% CI, 2.2–5.7) among those employed ≥ 10 years. As there was only one death attributed to asbestosis among those working < 1 year, 15 months was used as the cut point for the lowest category of exposure duration (providing a more stable estimator for comparison in the SRR analysis). Those working < 15 months were 38.2 (approximate 95% CI, 7.7–125.1) times more likely than expected to die from asbestosis; among those employed ≥ 10 years, the SMR was 628.6 (95% CI, 301.1–1185.1). The SRRs for lung cancer, nonmalignant respiratory disease, and asbestosis increased across increasing categories of exposure duration, and for each outcome, the test for a linear trend in the slope of the SRRs was statistically significant at the *p* < 0.05 level (Table 4).

## Discussion

Libby vermiculite workers experienced significant excess deaths from all causes, all cancers, lung cancer, cancer of the pleura, and asbestosis. Mortality from asbestosis and lung cancer increased with increasing cumulative exposure to airborne asbestos and other amphibole fibers.

Results of the present study are consistent with findings of previous mortality studies of workers from this cohort (Amandus and Wheeler 1987; McDonald et al. 1986, 2004). Amandus and Wheeler (1987) studied the mortality experience through 1981 of 575 white men with mean exposure estimated at 200 fibers/cc-years, who were hired at Libby before 1970 and worked at least 1 year. These researchers reported (Table 5) an overall SMR of 2.2 (95% CI, 1.4–3.4) for lung cancer (ICD-8 codes 162–163), with an SMR of 6.7 observed among those with ≥ 400 fiber-years cumulative exposure (not shown). However, the study did not have sufficient power to adequately assess lung cancer risk at lower exposure levels. The overall SMR for nonmalignant respiratory disease (ICD-8 codes 460–519) was 2.4 (95% CI, 1.5–3.8), but the small number of deaths through 1981 did not support clear conclusions about the exposure–response relationship.

Under contract with the company that operated the mine and mill from 1963–1990, McDonald et al. (1986) evaluated the mortality experience through mid-1983 of 406 white men with a mean cumulative exposure of 144.6 fibers/cc-years; these men had at least 1 year of tenure and were hired before 1963. McDonald et al. (2004) later independently reevaluated the mortality experience of these workers with follow-up through 1998. Overall SMRs of 2.4 (95% CI, 1.7–3.2) for respiratory cancer (44 deaths; ICD-9 codes 160–165) and 3.1 (95% CI, 2.3–4.1) for nonmalignant respiratory disease (51 deaths; ICD-9 codes 010–108 and 460–519) were reported (Table 5). Using Poisson regression, McDonald et al. (2004) found an exposure–response relationship between cumulative fiber exposure and respiratory cancer [highest quartile of cumulative exposure relative risk (RR) = 3.2; 95% CI, 1.2–8.8], nonmalignant respiratory disease (highest quartile RR = 3.1; 95% CI, 1.2–8.4), and mesothelioma (highest quartile RR = 3.4; 95% CI, 0.4–33.2). Asbestosis mortality per se was not described.

The summary SMRs for lung cancer and nonmalignant respiratory disease reported here are somewhat lower than those reported by McDonald et al. (2004), partially because my analysis used no tenure exclusion. More importantly, previous studies of Libby workers excluded those hired after the 1960s; the present study includes workers hired through 1981—that is, workers whose employment began after exposure intensity had been significantly reduced (Amandus et al. 1987). To assist in comparison between studies, summary SMRs for the subcohort of 864 white men (hired 1935–1981) who worked at least 1 year are included in Table 5.

However, the present analysis reveals substantial disease even among workers employed < 1 year. Short-term workers were 1.6 (95% CI, 1.1–2.1) times more likely to die from lung cancer and 2.1 (95% CI, 1.6–2.8) times more likely to die from nonmalignant respiratory disease than the comparable U.S. population (Table 4). Further, even among workers employed < 1 year, increasing cumulative fiber exposure was observed to predict nonmalignant respiratory disease mortality (not shown). Thus, including the mortality experience of workers employed < 1 year provides a more realistic picture of the true effect of working at Libby.

SMRs observed here for asbestos-exposed vermiculite workers are similar to those reported in other studies of asbestos-exposed workers. Goodman et al. (1999) conducted a meta-analysis of 69 asbestos-exposed cohorts (including a subset of the Libby cohort), and found a meta-SMR for lung cancer of 1.6 (95% CI, 1.6–1.7) after allowing for 10 years cancer latency. Honda et al. (2002) evaluated mortality among tremolite-exposed talc miners and millers, reporting an SMR of 2.2 (95% CI, 1.5–3.2) for nonmalignant respiratory disease, similar to the SMR of 2.4 (95% CI, 2.0–2.9) reported here among tremolite-exposed vermiculite workers.

The literature does not provide SMRs for asbestosis mortality among other tremolite asbestos–exposed cohorts. Among Libby workers, the SMRs for asbestosis were substantially higher than expected based on the U.S. population experience. The SMRs reported here may be inflated; that is, the small number of expected deaths from asbestosis (≤ 0.13) may have resulted in unstable estimators (Checkoway et al. 2004). On the other hand, the extremely high tremolite asbestos exposure (Amandus et al. 1987) these workers experienced (generally without respiratory protection), or concomitant exposure to other fibrous amphiboles, may have caused more disease than usually observed with less intense fiber exposure.

Clearance of asbestos fibers (and fiber toxicity) is believed to be a function of fiber size. Phagocytosis is limited by the size of human macrophages (generally 14–21 μm) (ATSDR 2003). Using standard asbestos fiber-counting methods (i.e., optical PCM considering only fibers ≥ 5 μm in length), 36% of fibers from Vermiculite Mountain had lengths > 20 μm, and 10% were > 40 μm (Amandus et al. 1987). Fibers longer than 20 μm have been associated with asbestosis. These long fibers, longer than the human macrophage, result in incomplete phagocytosis, perhaps partially explaining the unusually high mortality from asbestosis observed among the Libby workers.

Further, using transmission electron microscopy, around 65% of airborne fibers collected at Libby were found to be < 5 μm (ATSDR 2003). Animal and *in vitro* studies suggest that fibers < 5 μm may also play a role in fibrosis, particularly under conditions of overload. Intense exposures in early years and some jobs (mill sweeper, railroad car cleaner) may have resulted in overload, limiting clearance even of small fibers (ATSDR 2003). Thus, the high SMRs for asbestosis observed among Libby workers may be a function of fiber length and/or biopersistence.

The long-term biopersistence of the Libby fibers is supported by the work of Lockey and colleagues (Lockey et al. 1984; Rohs et al. 2005). In 1980, 4.4% of a cohort of 513 Ohio manufacturing workers exposed to expanded vermiculite and/or concentrate from Libby was found to have pleural changes documented on chest radiograph (Lockey et al. 1984). Rohs et al. (2005) reevaluated 236 of these workers > 20 years after the plant stopped using the asbestos-contaminated Libby vermiculite, and documented that 26% had pleural changes on chest radiograph. Further, an exposure–response effect was observed, with the proportion of workers with pleural changes rising from 5% among those in the lowest exposure group to 44% among workers with the heaviest exposure.

There has been no published human or animal (and very little *in vitro*) research on the potential health effects of winchite and richterite (Cleveland 1984; Collan et al. 1986; Holopainen et al. 1986), two unregulated amphibole minerals in the same mineralogic series as tremolite. However, another unregulated amphibole with similar elemental composition and structure has been linked with asbestos-related mortality—mesothelioma in a community exposed to fluoroedenite (Comba et al. 2003). Although speculative, it is possible that Libby workers experienced effects from two or more of the amphibole minerals at Vermiculite Mountain (tremolite, winchite, richterite), or from an amphibole and quartz or mica, and that these joint effects may have contributed to the extreme asbestosis SMRs. Quartz is known to cause silicosis (another pneumoconiosis), although only one worker in this cohort died from silicosis. Vermiculite belongs to the mica family; mica has previously been linked with pneumoconiosis (Skulberg et al. 1985; Venter et al. 2004; Zinman et al. 2002).

It is also possible that pneumoconiosis resulting from exposure to these other minerals may have been misclassified as asbestosis on death certificates. Local physicians were aware that Libby vermiculite workers were exposed to asbestos, and this exposure was sometimes mentioned on death certificates. Alternatively, these elevated SMRs may reflect the joint effect of lifelong ambient exposure to asbestos fibers from living in the nearby town combined with high intensity fiber exposure at work. In any case, a clear relationship between increasing cumulative fiber exposure and increasing asbestosis mortality was observed.

### Limitations

Retrospective exposure estimates were developed using a combination of government inspection reports, company compliance-monitoring data (available from 1974), and professional judgment (Amandus et al. 1987; McDonald et al. 1986). These methods likely resulted in some measurement error. Assuming that any misclassification of exposure was not systematic, the most likely effect is bias toward the null (Checkoway et al. 2004; Mannetje et al. 2002). Further, there is insufficient sampling data to develop reliable exposure estimates for potential confounders for lung cancer such as workplace exposure to diesel particulate generated by mine machinery, or exposure to respirable crystalline silica dust.

Work history data were missing for 9% of workers who terminated before 1954. These workers were assumed to have worked about 1 year, based on the average employment duration among other workers who terminated between 1935 and 1953. Without the missing data, it is not possible to determine with certainty the impact of this approach, although the most likely effect is bias toward the null. Analysis deleting the 55 workers with missing termination dates did not result in appreciably different effect estimates than are reported here.

No minimum employment duration restriction was imposed in the present analysis. Thus, overall effect estimates are somewhat lower than those reported in previous studies of this cohort (Table 5). Elevated SMRs were observed for the occupational respiratory diseases of interest and among those employed < 1 year. The assumption that biologically significant exposures occurred immediately at hire is realistic in this workplace (particularly in early years), where new hires were frequently assigned to the labor pool and often rotated through the most heavily exposed jobs. Evaluation of job-assignment patterns suggests that some job tasks (i.e., “bin mucker”) were systematically assigned to transitory workers. Alternatively, these jobs may have been so onerous that newly hired workers quit after only a day or two.

### Strengths

Strengths of this study include the long period of vital status follow-up—> 65 years from 1935–2001. A minimum of 20 years since first exposure to the end of follow-up allowed sufficient latency for most cancers. Previously published analyses of data from this occupational cohort reported SMRs for nonmalignant respiratory disease (Amandus and Wheeler 1987; McDonald et al. 1986, 2004). In the analysis reported here I made use of comparison rates for asbestosis that have become widely available within the last 10 years. The resulting effect estimates for asbestosis provide a more accurate description of the magnitude of asbestos-related disease among this cohort of workers.

## Conclusions

Significant elevations in SMRs for asbestosis, lung cancer, and cancer of the pleura were observed among Libby vermiculite workers. Exposure–response relationships were noted for asbestosis and lung cancer. Significant excess mortality from nonmalignant respiratory disease was observed even among workers with cumulative exposure < 4.5 fibers/cc-years [i.e., a worker’s cumulative lifetime exposure, if exposed to asbestos fibers at the current OSHA standard of 0.1 fibers/cc over a 45-year working life (U.S. Department of Labor 2006)]. Since vermiculite from the Libby mine was used to make loose-fill attic insulation that remains in millions of homes, these findings highlight the need for better understanding and control of exposures that currently occur when homeowners or construction renovation workers (including plumbers, cable installers, electricians, telephone repair personnel, and insulators) disturb loose-fill attic insulation made with asbestos-contaminated vermiculite from Libby, Montana.

## Figures and Tables

**Table 1:** Demographic and exposure characteristics of 1,672 white male Libby, Montana, vermiculite workers hired during 1935–1981 by multiple^a^ cause of death.

Characteristic	All workers	All deaths	Lung cancer^a^	Mesothelioma^a^	Asbestosis^a^
No. of workers	1,672	767	99	15	40
Mean year of birth	1930	1917	1921	1926	1919
Mean year of hire	1959	1952	1953	1955	1952
Mean year of death	—	1984	1986	1989	1988
Mean age at hire (years)	29.7^b^	34.5	32.1	29.3	32.4
Mean age at death (years)	—	67.0	64.7	63.6	69.1
Mean person-years of follow-up (no lag)	34.8^c^	32.5	32.7	34.3	36.7
Mean employment duration (years)^d^	4.0	5.0	7.1	10.8	14.6
Worked < 1 year (*n* = 808)^e^	0.25	0.22	0.21	—e	0.55
Worked ≥ 1 year (*n* = 809)^d^	7.7	9.6	12.4	11.6	15.7
Median cumulative exposure (fibers/cc-years)^d^	8.7	21.0	28.2	145.1	228.4
Worked < 1 year (*n* = 808)	2.6	5.8	6.1	—e	36.2
Worked ≥ 1 year (*n* = 809)^d^	43.4	135.1	124.5	146.4	244.8

a Includes any mention of condition on death certificate.

b Mean age at hire was significantly lower among study subjects who worked < 1 year compared with those who worked longer (28.9 vs. 30.4; *p* = 0.0016).

c Totaling 58,186 person-years of follow-up without exposure lag.

d Fifty-five workers with unknown termination date were excluded when calculating mean duration of employment and median cumulative exposure.

e Among study subjects who worked < 1 year, there were 42 lung cancer deaths, 1 mesothelioma death, and 3 asbestosis deaths.

**Table 2:** SMRs for selected occupational respiratory diseases among 1,672 white male Libby, Montana, vermiculite workers by underlying cause of death (1960–2001).^a^,^b^

		Deaths		
Cause of death	ICD-9 codes	Obs	Exp	SMR^c^ (95% CI)
All causes		711	574.04	1.2 (1.1–1.3)
All cancer	140–239, 273.1, 273.3	202	147.58	1.4 (1.2–1.6)
Cancer of the trachea, bronchus, or lung	162	89	52.53	1.7 (1.4–2.1)
Possible mesothelioma^d^				
Mesothelioma (1999–2001)	C45 (ICD-10)^d^	2	0.13	15.1 (1.8–54.4)
Cancer of the pleura	163	4	0.17	23.3 (6.3–59.5)
Cancer of unspecified sites	160, 164–165, 187, 194–199	25	10.29	2.4 (1.6–3.6)
Connective tissue cancer (1940–2001)^e^	171	4	0.85	4.7 (1.3–12.0)
Nonmalignant respiratory disease	460–519	111	46.70	2.4 (2.0–2.9)
Asbestosis	501	22	0.13	165.8 (103.9–251.1)
Chronic obstructive pulmonary disease	490–492, 496	53	23.81	2.2 (1.7–2.9)
Other nonmalignant respiratory diseases	470–478, 494–495, 504, 506–519	19	7.09	2.7 (1.6–4.2)

Abbreviations: Exp, expected; Obs, observed.

a Analysis based on a 15-year exposure lag with 32,021 person-years of follow-up.

b For clarity, only respiratory causes of death that were elevated compared with the U.S. white male population are included.

c Comparison for SMR is deaths in U.S. population of same age category, race, and sex during same calendar time period.

d Before 1999, when a unique ICD-10 code was assigned to mesothelioma, mesothelioma deaths were coded to other causes such as cancer of the pleura or cancer of unspecified sites.

e Because small numbers result in unstable estimators, the SMR for connective tissue cancer is reported for deaths 1940–2001, with no exposure lag.

**Table 3:** SMRs and SRRs for selected occupational respiratory diseases among 1,672 Libby, Montana, vermiculite workers by underlying cause of death (1960–2001) and increasing level of cumulative exposure.^a^

			No. of deaths			
Cause of death	Cumulative exposure (fibers/cc-years)	Person-years	Obs	Exp	SMR^b^ (approximate 95% CI)	SRR^c^ (95% CI)
Lung cancer	0.0–4.49	10,400	19	13.02	1.5 (0.9–2.3)	1.0 (—)^c^
	4.5–22.9	9,207	24	14.62	1.6 (1.1–2.5)	1.1 (0.6–2.0)
	23.0–99.9	6,667	23	12.95	1.8 (1.1–2.7)	1.4 (0.7–2.7)
	≥ 100.0	5,748	23	11.93	1.9 (1.2–2.9)	1.5^d^ (0.8–2.8)
NMRD	0.0–4.49	10,400	18	10.20	1.8 (1.1–2.8)	1.0 (—)^c^
	4.5–19.9	8,465	24	12.20	2.0 (1.3–3.0)	1.2 (0.6–2.3)
	20.0–84.9	6,725	26	11.69	2.2 (1.5–3.3)	1.5 (0.8–2.9)
	85.0–299.9	4,357	20	7.85	2.6 (1.6–4.0)	1.4 (0.7–2.7)
	≥ 300.0	2,075	23	4.76	4.8 (3.1–7.3)	2.8^e^ (1.3–5.7)
Asbestosis	0.0–49.9	22,341	3	0.08	37.3 (7.5–122.3)	1.0 (—)^c^
	50.0–249.9	7,136	8	0.04	212.6 (91.6–433.2)	7.3 (1.9–28.5)
	≥ 250.0	2,544	11	0.01	749.1 (373.0–1367.8)	25.3^f^ (6.6–96.3)

Abbreviations: Exp, expected; NMRD, nonmalignant respiratory disease; Obs, observed.

a Analysis based on a 15-year exposure lag with 32,021 person-years of follow-up.

b Comparison for SMR is deaths in U.S. population of same age category, race, and sex during same calendar time period.

c Comparison for SRR is the lowest exposure group, with the SRR fixed at 1.0.

d Test for a linear trend in the slope of the SRRs, testing the hypothesis that β_1_ = 0 against the alternative hypothesis that cumulative fiber exposure predicts lung cancer mortality: slope = 5.479 × 10^−6^; SE = 1.574 × 10^−6^; 95% CI for slope, 2.393 × 10^−6^ to 8.564 × 10^−6^. Because the 95% CI for the slope does not include 0, the hypothesis was rejected in favor of the alternative; χ^2^ = 12.11; *p* < 0.001.

e Test for a linear trend in the slope of the SRRs for nonmalignant respiratory disease mortality: slope = 5.004 × 10^−6^; SE = 1.907 × 10^−6^; 95% CI for slope, 1.267 × 10^−6^ to 8.741 × 10^−6^; χ^2^ = 6.89; *p* < 0.01.

f Test for a linear trend in the slope of the SRRs for asbestosis mortality: slope = 5.479 × 10^−6^; SE = 8.985 × 10^−7^; 95% CI for slope, 3.718 × 10^−6^ to 7.24 × 10^−6^; χ^2^ = 37.18; *p* < 0.001.

**Table 4:** SMRs and SRRs for selected occupational respiratory diseases among 1,672 Libby, Montana, vermiculite workers by underlying cause of death (1960–2001) and duration of exposure.^a^

			No. of deaths			
Cause of death	Exposure duration	Person-years	Obs	Exp	SMR (approximate 95% CI)^b^	SRR (95% CI)^c^
Lung cancer	< 1 year	16,742	41	26.29	1.6 (1.1–2.1)	1.0 (—)^c^
	1–9.9 years	13,047	34	20.64	1.7 (1.1–2.3)	1.1 (0.7–1.8)
	≥ 10 years	2,232	14	5.59	2.5 (1.4–4.3)	1.8 (0.9–3.4)^d^
NMRD	< 1 year	16,742	48	22.83	2.1 (1.6–2.8)	1.0 (—)^c^
	1–9.9 years	13,047	44	18.65	2.4 (1.7–3.2)	1.2 (0.8–2.0)
	≥ 10 years	2,232	19	5.22	3.6 (2.2–5.7)	1.4 (0.8–2.3)^e^
Asbestosis	< 15 months	19,152	3	0.08	38.2 (7.7–125.1)	1.0 (—)^c^
	15 months–9.9 years	10,637	9	0.04	236.0 (107.8–461.1)	6.7 (1.8–24.9)
	≥ 10 years	2,232	10	0.02	628.6 (301.1–1185.1)	17.5 (4.7–64.5)^f^

Abbreviations: Exp, expected; NMRD, nonmalignant respiratory disease; Obs, observed.

a Analysis based on a 15-year exposure lag with 32,021 person-years of follow-up.

b Comparison for SMR is deaths in U.S. population of same age category, race, and sex during same calendar time period.

c Comparison for SRR is the lowest exposure group, with the SRR fixed at 1.0.

d Test for a linear trend in the slope of the SRRs, testing the hypothesis that β_1_ = 0 against the alternative hypothesis that duration of exposure predicts lung cancer mortality: slope = 0.0302; SE = 0.0118; 95% CI for slope, 0.007–0.0534. Because the 95% CI for the slope does not include 0, the hypothesis was rejected in favor of the alternative; χ^2^ = 6.50; *p* < 0.05.

e Test for a linear trend in the slope of the SRRs for nonmalignant respiratory disease mortality: slope = 0.0226; SE = 0.0073; 95% CI for slope, 0.0083–0.0368; χ^2^ = 9.64; *p* < 0.01.

f Test for a linear trend in the slope of the SRRs for asbestosis mortality: slope = 0.0431; SE = 0.0002; 95% CI for slope, 0.0427–0.0435; χ^2^ = 4.69 × 10^4^; *p* < 0.001.

**Table 5:** Cohort characteristics and SMRs from several studies of Libby, Montana, vermiculite workers.

				Present study	
	McDonald et al. (1986, 2004) (worked at least 1 year)^a^		Amandus and Wheeler (1987) (worked at least 1 year)^b^	Worked at least 1 day^c^,^d^	Worked at least 1 year^d^,^e^
Cause of death	Deaths to mid-1983 (SMR)^f^	Deaths through 1998 [SMR (95% CI)]	Deaths through 1981 [SMR (95% CI)]	Deaths 1960–2001 [SMR (95% CI)]	Deaths 1960–2001[SMR (95% CI)]
All causes	1.2^g^	1.3 (1.1–1.4)	1.1 (0.9–1.3)	1.2 (1.1–1.3)	1.3 (1.2–1.4)
All cancer	—h	— (—)	1.3 (0.9–1.8)	1.4 (1.2–1.6)	1.6 (1.3–1.9)
Respiratory cancer	2.5	2.4 (1.7–3.2)	— (—)	1.7 (1.4–2.1)	2.0 (1.5–2.5)
Lung cancer	—	— (—)	2.2 (1.4–3.4)	1.7 (1.4–2.1)	1.9 (1.4–2.5)
NMRD	2.6	3.1 (2.3–4.1)	2.4 (1.5–3.8)	2.4 (2.0–2.9)	2.6 (2.0–3.4)
Asbestosis	—	— (—)	— (—)	165.8 (103.9–251.1)	307.0 (189.9–469.2)

NMRD, nonmalignant respiratory disease.

a Study of 406 white men who were hired before 1963 (mean duration of employment = 8.7 years; mean cumulative exposure estimate = 144.6 fibers/cc-years; total person-years not provided in the publications).

b Study of 575 white men who were hired before 1970 (mean duration of employment = 8.3 years; mean cumulative exposure estimate = 200 fibers/cc-years; 13,502 person-years of follow-up.

c Included 1,672 white men who were hired 1935–1981 (mean duration of employment = 4.0 years; mean cumulative exposure estimate = 96.3 fibers/cc-years; person-years in 15-year lagged analysis = 32,021).

d Fifty-five workers with date of termination unknown were excluded in calculating the mean duration of employment and estimating the mean cumulative exposure.

e Included 864 white men who were hired 1935–1981 (mean duration of employment = 7.7 years; mean cumulative exposure estimate = 184.0 fibers/cc-years; person years in 15-year lagged analysis 16,030).

f Comparison for SMR is deaths among white men in U.S. population of same age category during the same calendar time period.

g 95% CIs not presented in article.

h Estimator not presented in article.
